# A Retrospective, Pilot Study of De Novo Antidepressant Medication Initiation in Intensive Care Unit Patients and Post-ICU Depression

**DOI:** 10.1155/2017/5804860

**Published:** 2017-09-13

**Authors:** Daniel Haines, Johanna Hild, Jianghua He, Lucy Stun, Angie Ballew, Justin L. Green, Lewis Satterwhite, Brigid C. Flynn

**Affiliations:** ^1^Department of Anesthesiology, University of Kansas Hospital, Kansas City, KS, USA; ^2^Department of Biostatistics, University of Kansas Hospital, Kansas City, KS, USA; ^3^Department of Pharmacy, University of Kansas Hospital, Kansas City, KS, USA; ^4^Department of Surgery, University of Kansas Hospital, Kansas City, KS, USA; ^5^Department of Medicine, University of Kansas Hospital, Kansas City, KS, USA

## Abstract

Post-ICU Syndromes (PICS) remain a devastating problem for intensive care unit (ICU) survivors. It is currently unknown if de novo initiation of an antidepressant medication during ICU stay decreases the prevalence of post-ICU depression. We performed a retrospective, pilot study evaluating patients who had not previously been on an antidepressant medication and who were started on an antidepressant while in the trauma surgical, cardiothoracic, or medical intensive care unit (ICU). The PHQ-2 depression scale was used to ascertain the presence of depression after ICU discharge and compared this to historical controls. Of 2,988 patients admitted to the ICU, 69 patients had de novo initiation of an antidepressant medication and 27 patients were alive and available for study inclusion. We found the prevalence of depression in these patients to be 26%, which is not statistically different than the prevalence of post-ICU depression in historical controls [95% CI (27.6%, 51.6%)]. De novo initiation of an antidepressant medication did not substantially decrease the prevalence of post-ICU depression in this retrospective, pilot study.

## 1. Introduction

Post-intensive care syndrome (PICS) is a problem that has been in existence since the inception of intensive care units (ICU). Recently, more focus has been placed on this syndrome as new approaches are being developed to diagnose and to treat this problem. One factor in the reported increase in PICS is the continued growth in ICU care. In recent years the number of ICU beds in the United States has increased by 15% [1]. Survival rates in patients admitted to adult ICU average 71–90%, depending on age and severity of illness [2]. This leads to many ICU survivors who may be left traumatized by their ICU experience, a condition known as PICS.

PICS is a syndrome lacking specific diagnostic criteria. However, the range of morbidities encompassed by PICS includes three broad categories: physical impairment, cognitive impairment, and psychiatric impairment, such as posttraumatic stress disorder (PTSD) [3]. PICS can also have detrimental effects on family members, such as care burdens, financial implications, and psychological issues, a condition termed PICS-F [4]. PICS can be a devastating morbidity that accompanies ICU survival. The prevalence of PTSD, which can be a part of PICS, following ICU admission is 5–64% [5]. Furthermore, PICS remains in about 24% of patients after an average of eight years after ICU discharge [6].

Currently, there are no proven therapies to combat PICS while in the ICU. We sought to elucidate in a heterogeneous ICU population (1) whether the initiation of an antidepressant medication while in the ICU affected the prevalence of post-ICU depression and (2) whether patients remained on these antidepressants after discharge.

## 2. Materials and Methods

After institutional review board approval, electronic medical record review of patient charts identified patients who had been started on an antidepressant medication while being hospitalized in the trauma surgery and medical and cardiothoracic intensive care units (ICU) from October 1, 2015, to January 1, 2017. The initiation was at the discretion of the intensivist with no standardized approach dictated by this study. Data was not ascertained for specific reason behind the intensivist utilization of an antidepressant medication. The search specifically filtered drugs that had an indication for the treatment of depression according to Lexicomp Clinical Drug Information [7]. From there, all benzodiazepines and atypical antipsychotics were excluded as these drugs are most commonly initiated for reasons other than depression in the ICU setting. Drugs that could be used for depression were excluded if the indication for prescription was not depression. For example, patients on an antidepressant medication for enuresis or restless leg syndrome were excluded. Patients who had been on an antidepressant prior to hospitalization were excluded.  Table 1 displays the classes of antidepressant medications initiated in the study population.

Patients who met study criteria were first contacted by a letter in February 2017 stating that they were being included in a study on depression following ICU stay and given the option to decline participation prior to the telephone interview. No patients declined at this point. Study patients were telephoned in February-March 2017 and asked the following:

Are you still taking your (insert name of antidepressant initiated in ICU) or are you taking another antidepressant?

The percentage of patients still taking their ICU prescribed antidepressant medication and/or switched medications was tabulated. Following this question, the prevalence of depression was calculated by administering the PHQ-2 depression scale (Table 2) [8]. The median time range to phone call follow-up after ICU stay was 8.5 months [2, 12].

The prevalence of post-ICU depression was ascertained via historical controls based on recent, large systematic review and analysis using PubMed, EMBASE, Cumulative Index of Nursing and Allied Health Literature, PsycINFO, and Cochrane Controlled Trials Registry (1970–2015) [9].

## 3. Results

A total of 2,988 patients were admitted to the trauma surgery, medical, and cardiothoracic ICU at our tertiary care hospital during the study period. Of those, 69 patients were retrospectively identified via electronic medical record review as having been initiated on an antidepressant medication during their ICU stay: three patients from the cardiothoracic ICU, 20 patients from the medical ICU, and 46 patients from the trauma surgical ICU. Of those, 18 patients had died at the time of follow-up in February-March 2017. Twenty-three patients were unable to be contacted by telephone and one declined to participate.

Of the 27 study patients, 24 were trauma surgical ICU patients, three were cardiothoracic ICU patients, and none were medical ICU patients. Telephone interviews revealed that 26% (7/27) of patients were depressed [95% CI (27.6%, 51.6%)]. Notably, 74% (20/27) of the post-ICU patients did not suffer from depression.

**Figure 1:**
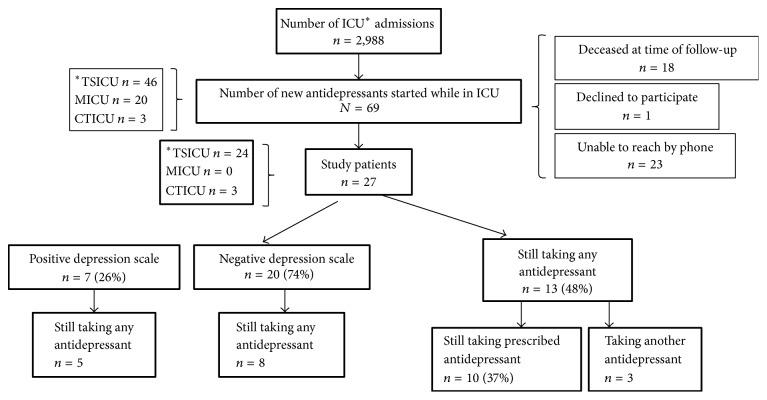
Graphical representation of patients included and excluded from the study and outcomes. ^*∗*^TSICU = trauma surgical intensive care unit; MICU = medical intensive care unit; CTICU = cardiothoracic intensive care unit.

Of the 27 study patients, 48% (13/27) were still taking an antidepressant medication with the majority (*n* = 10) still taking the prescribed antidepressant initiated during ICU admission. Three patients had been switched to another antidepressant and one patient had an additional antidepressant added to the prescribed antidepressant from the ICU (Figure 1). When analyzing the subset of patients who were found to be depressed, the majority remained on their ICU prescribed antidepressant with one patient having an additional antidepressant added (*n* = 5/7). Of the nondepressed patients, less than half (*n* = 8/20) remained on an antidepressant.

## 4. Discussion

The present study found a prevalence of depression in 26% of a subset of ICU patients who were initiated on an antidepressant in the ICU. A recent review and meta-analysis found the prevalence of post-ICU depression to be 29–34% within the first year after ICU discharge in patients regardless of medication utilization [9]. Thus antidepressant initiation while in the ICU did not seem to decrease the prevalence of post-ICU depression [95% CI (27.6%, 51.6%)]. Although the prevalence in this study of 26% is lower than the previous studies (29–34%), the difference is not substantial from the clinical aspect and the difference is not statistically significant either as the 95% CI found in this study covers these previous rates. Thus, this retrospective, pilot study, which is the first of its kind, does not provide evidence that initiation of an antidepressant during ICU stay has any obvious effect in decreasing the prevalence of depression after discharge.

Initiation of antidepressant medications during ICU stay in trauma surgical, medical, and cardiothoracic ICU patients is not common at our institution. Of the 2,988 admissions to these ICU, only 2% (*n* = 69) of patients were started on an antidepressant. Clinicians may be hesitant to do so because there is no research concerning this therapy in decreasing depression either while in the ICU or afterwards. In fact, the present study is the first to analyze the intervention of antidepressant initiation in ICU patients and subsequent effect on post-ICU depression.

The PHQ-2 depression scale (Table 2) has been validated in various populations, including trauma ICU patients [8, 10]. In this scale, a score ≥ 3 (total possible score of 0–6 points) correlates with depression. There are many advantages to this depression scale, such as the simplicity for providers to administer and for patients to complete. The PHQ-2 has a likelihood for diagnosing major depression nearly identical to the overall likelihood reported for nine other depression case finding instruments [11]. Research has shown that a single question about depressed mood has a sensitivity of 85–90% for major depression [12, 13] and adding a second question about anhedonia increases the sensitivity to 97% with a specificity of 67% [14].

Most antidepressants take two to eight weeks to demonstrate antidepressant effects, although a placebo effect may be seen prior to this [15, 16]. Despite the knowledge that the antidepressant effect will occur at some point, even post-ICU discharge, clinicians may still have reluctance to initiate these drugs in an ICU setting due to side effects like cardiac conduction disturbances, sedative effects, risk of serotonin syndrome, especially with concomitant linezolid antibiotic [17], potential for increased bleeding [18, 19], and other side effects such as dry mouth and blurred vision. Indeed, this intervention did not demonstrate a substantial change in the prevalence of post-ICU depression in our study based on historical cohorts; thus these risks may outweigh any potential benefits of antidepressant initiation in the ICU.

Until further research, starting antidepressant medications in the ICU does not seem to be a highly promising therapy for the prevention of post-ICU depression. There have been other nonmedicinal therapies trialed in ICU patients in hopes of alleviating PICS such as the ICU Liberation ABCDEFGH bundle which focuses on pain, agitation, delirium, and movement [20]. However, there is currently limited evidence concerning the benefits of this therapy and PICS reduction.

When examining the benefit of post-ICU interventions aimed at reducing PICS, data is not robust [9]. Four studies utilizing physical rehabilitation [21, 22] and use of a prospective ICU diary have shown a decrease in depression [23] and PTSD [24, 25]. Other post-ICU discharge follow-up interventions have not shown substantial benefit [26, 27]. Current widespread initiatives focused on supporting survivors include THRIVE from the Society of Critical Care Medicine [28] and InS:PIRE which stands for Intensive Care Syndrome: Providing Independence and Return to Employment [29].

Newer research is focusing on the phenomenon of reconsolidation of memories with medication [30]. The discovery that memories are not fixed, but malleable to change, may benefit ICU survivors. Specifically, propranolol has been shown to prevent stressful memories and transmission of stress hormones linked to PTSD. By dissociating the state of sympathetic arousal from the memory, propranolol may be a promising therapy for PICS. The effects of propranolol both are immediate and can last longer than two years if propranolol is taken immediately prior to the traumatic event.

*Limitations.* Since PICS is a known, major problem in critical care, this exploratory study was undertaken as the first to look at the initiation of antidepressant medications in ICU patients. The current study was undertaken as a preliminary pilot study and was undertaken in a retrospective nature. Depression scales were not given to patients prior to ICU admission; thus some may have had untreated and undiagnosed depression.

Secondly, we did not measure the prevalence in post-ICU depression in all patients admitted to the ICU who were not started on antidepressants but instead used historical controls in previous studies as our comparison group. Some of the historical controls may have been on antidepressant medications as previous studies have not looked at this.

Thirdly, the definition of antidepressant used in this study was very strict. We excluded benzodiazepines and atypical antipsychotics that some patients may use for depression. These were excluded in order to focus on depressive symptoms, but patients who found reduction in depression with these agents while in the ICU and after discharge may have been unfortunately excluded.

Next, the follow-up time was discrepant among the patients in this study. The amount of time passing from postdischarge from the ICU to participation in the study ranged from 2 to 12 months with a median of 8.5 months. This may have impacted the propensity of a patient to remain on their prescribed medication and to encounter depressive symptoms. However, it has been shown that patients with post-ICU depression tend to display depressive symptoms uniformly at 2-3 months, 6 months, and 12 months after ICU discharge [9].

Lastly, due to the low rate of de novo initiation of antidepressants in our population, we were unable to show statistical significance of our findings. A sample size of 31 patients is not large enough to provide testing power. A larger, multicenter study should be undertaken to confirm whether the prevalence is statistically different.

## 5. Conclusion

De novo initiation of antidepressant medications while in the ICU did not substantially decrease the prevalence of postdischarge depression which remained similar to historical controls at 26%. Of the patients with postdischarge depression, most remained on their antidepressant medication. Based on these exploratory results, initiating antidepressants in ICU patients with the primary aim of decreasing postdischarge depression is not warranted. Larger trials could help identify the potential of such a strategy. In the meantime, focus should continue on minimally invasive ICU bundles and the postdischarge initiatives that may prevent and/or treat PICS.

## Figures and Tables

**Table 1:** Classes of antidepressant medications initiated in ICU patients.

Antidepressant class	Number of patients (*n* = 27)
SARI	15
SSRI	6
NaSSA	3
TCA	2
SNRI	1

SARI = serotonin antagonist and reuptake inhibitor; SSRI = selective serotonin reuptake inhibitors; NaSSA = noradrenergic and specific serotonergic antidepressant; TCA = tricyclic antidepressant; SNRI = serotonin-norepinephrine reuptake inhibitors.

**Table 2:** The Patient Health Questionnaire-2 (PHQ-2).

Over the past 2 weeks, how often have you been bothered by any of the following problems?	Not at all	Several days	More than half of the days	Nearly every day
(1) Feeling down, depressed, or hopeless	0	1	2	3
(2) Little interest or pleasure in doing things	0	1	2	3
